# Distinct thalamic volumetric profiles in cervical and upper limb dystonia

**DOI:** 10.1055/s-0046-1825521

**Published:** 2026-07-21

**Authors:** Hüseyin Yiğit, Abdulkerim Gökoğlu

**Affiliations:** 1 Cappadocia University, Vocational Health School, Nevşehir, Türkiye.; 2 Konya State Educating & Teaching Hospital, Department of Neurosurgery, Konya, Türkiye.

**Keywords:** Torticollis, Magnetic Resonance Imaging, Thalamus, Dystonia, Neuroimaging

## Abstract

**Background:**

Dystonia is a large-scale motor network disorder involving the basal ganglia, cerebellum, and thalamus.

**Objective:**

This study investigated the volumetric characteristics of thalamic subnuclei in patients with isolated cervical (CD) and upper limb (ULD) dystonias, and their relationship with disease severity.

**Methods:**

We analyzed high-resolution T1-weighted MR-images from the OpenNeuro database. The sample included 37 healthy controls, 25 CD, and 29 ULD patients. Thalamic nuclei were automatically segmented using the DeepThalamus pipeline. Group differences were analyzed using analysis of covariance (ANCOVA), controlling for age, sex, and total intracranial volume (TIV). Relationships between disease severity and volumetry were assessed using Spearman's correlation.

**Results:**

A volumetric reduction trend was observed in the CD group compared with controls, particularly in the left lateral geniculate, right ventral lateral posterior, and right intermediate nuclei. Although statistical significance remained borderline after FDR correction ( *p* = 0.078), high Cohen's values (d > 1.4) were detected. In contrast, thalamic volumes were largely preserved in the ULD group. Correlation analyses showed that increased disease severity was associated with decreased volumes of the mammillothalamic tract in the CD group and the anterior ventral nuclei in the ULD group.

**Conclusion:**

Observed volumetric trends may provide preliminary insights into the role of specific thalamic substructures in cervical dystonia pathophysiology. Further studies in larger cohorts, supported by functional and diffusion imaging, are needed to clarify these findings.

## INTRODUCTION

Dystonia is a hyperkinetic movement disorder characterized by continuous or intermittent muscle contractions that cause abnormal postures or repetitive movements. 1 Among the most common forms of adult-onset isolated focal dystonias are cervical dystonia (CD) and upper limb dystonia (ULD). 2 While the pathophysiology of the disease has long been attributed to basal ganglia dysfunction, current data suggest that dystonia is a large-scale motor network disorder involving the basal ganglia, cerebellum, thalamus, and sensorimotor cortex. 3 4 5

Within this motor network, the thalamus serves as a critical “relay” station and integrator in relaying information from both the basal ganglia (pallidal inputs) and the cerebellum to the cortex. The ventral lateral (VL) thalamic nuclei (VLa and VLp), in particular, are known to play a central role in modulating movement and in the pathogenesis of movement disorders like dystonia due to their connections with the motor cortex. 6 Neurophysiological studies have shown changes in thalamic neuronal activity during dystonic movements and demonstrated that thalamic lesions or deep brain stimulation can alleviate dystonic symptoms. 7

Structural neuroimaging studies have reported abnormalities in both gray and white matter in idiopathic dystonia. Recent research suggests that different neuroanatomical profiles may be exhibited by subtypes of focal dystonia (such as CD and ULD). Paulo et al. 8 identified a striatal compartment (matrix and striosome) imbalance in ULD patients, while observing more widespread subcortical atrophy and white matter changes in CD patients. Similarly, Xu et al. 9 have demonstrated a correlation between this atrophy and thalamocortical white matter degeneration in idiopathic dystonia, indicating structural deterioration of the thalamus.

However, the thalamus is not a homogeneous structure; it consists of numerous subnuclei that are anatomically and functionally specialized for motor, sensory, and limbic functions. Previous studies have often treated the thalamus as a single entity, potentially overlooking volumetric changes in specific nuclei. Recent research 10 on patients with blepharospasm and oromandibular dystonia has demonstrated that thalamic nuclei (such as the pulvinar, lateral geniculate, and VL nuclei) may exhibit distinct atrophy patterns based on the disease phenotype. Nevertheless, studies comparing the specific involvement patterns of thalamic nuclei in different subtypes of focal dystonia, like CD and ULD, and their relationship with clinical severity is limited.

In this study, we aimed to investigate the volumetric characteristics of the thalamic nuclei in patients with isolated CD and ULD based on the dataset of Paulo et al. 8 Our hypothesis suggests that different clinical phenotypes may be associated with distinct atrophy patterns in the motor and sensory/limbic nuclei within the thalamus. Furthermore, we aimed to explore the correlation of these structural changes with the Burke-Fahn-Marsden Dystonia Rating Scale (BFMDRS) scores to investigate the relationship between volumetric changes in thalamic substructures and dystonia pathophysiology.

## METHODS

### Study group and data set

The original study protocol for data collection was approved by the Institutional Review Board (IRB) of Hospital Israelita Albert Einstein and the ethics committees of the participating centers (Hospital do Servidor Público Estadual and Universidade Federal de São Paulo). Written informed consent was obtained from all participants in accordance with the principles of the Declaration of Helsinki prior to data collection procedures. Since the current study involves the secondary analysis of deidentified data publicly shared in the OpenNeuro repository (Accession Number: ds006395), 11 it is exempt from local ethics committee approval.

The study sample consists of a total of 91 participants divided into three groups based on neurological and psychiatric evaluations:

- Healthy controls (HC, n = 37): Individuals aged 18 to 78 years with no history of neurological or psychiatric conditions.
- Cervical dystonia (n = 25): Patients diagnosed with isolated idiopathic CD.
- Upper limb dystonia (n = 29): Patients diagnosed with isolated idiopathic ULD.

It was confirmed that all included participants were right-handed. Disease severity in patients was assessed using the BFMDRS. Detailed demographic and clinical characteristics of the cohort are available in the original study by Paulo et al. 8

### Data collection procedure

Neuroimaging data were acquired using a MAGNETOM Prisma 3.0 T (Siemens Healthineers) magnetic resonance imaging (MRI) scanner equipped with a 64-channel head coil at Hospital Israelita Albert Einstein.

In this volumetric study, high-resolution T1-weighted anatomical scans from the dataset were utilized. While the original imaging protocol included T1-weighted, diffusion-weighted imaging (DWI), and functional MRI (fMRI) sequences, the automated segmentation processes in this study were performed solely on structural T1 images. These were acquired using a Magnetization-Prepared Rapid Gradient-Echo (MPRAGE) sequence with the following parameters: Repetition Time (TR) = 2,500 ms, Echo Time (TE) = 3.47 ms, Flip Angle = 7°, Inversion Time (TI) = 1,100 ms, and voxel size = 1.0 × 0.5 × 0.5 mm.

### Image processing and volumetry

The T1-weighted MR data were processed using the volBrain automated MR brain analysis platform ( https://volbrain.net ) to enable precise and reproducible measurement of brain structures, along with a three-dimensional (3D) slicer extension (SlicerVolBrain).

For the specialized segmentation of thalamic nuclei, the DeepThalamus pipeline, a novel deep learning method trained on ultra-high-resolution multimodal MRI data, was employed. The pipeline includes the following processing steps:

- Denoising of images using a spatially adaptive nonlocal means filter,
- Affine registration to MNI152 space,
- N4 bias field correction to remove inhomogeneities,
- Super-resolution using deep learning-based algorithms to upsample images to 0.5 mm isotropic resolution, and
- Segmentation of the thalamus into 13 subnuclei (26 bilateral structures in total) using a deep pyramidal network architecture.

Within the scope of this study, volumes (cm ^3^ ) were calculated for the following regions: anterior ventral (AVN), ventral anterior (VAN), ventral lateral anterior (VLa), ventral lateral posterior (VLp), ventral posterolateral (VPL), pulvinar (PN), lateral geniculate (LGN), medial geniculate (MGN), centromedian (CN), mediodorsal (MN), habenular (Hb), mammillothalamic tract (MTT), and intermediate space (ISN). 12

All thalamic subnucleus segmentations automatically generated by the DeepThalamus pipeline were visually checked one by one for anatomical accuracy by an expert neuroanatomist and neurosurgeon, as presented in the Supplementary Material ( https://www.arquivosdeneuropsiquiatria.org/wp-content/uploads/2026/03/ANP-2025.0475-Supplementary-Material.docx ). 13 No segmentation errors or artefacts were detected, so no participants were excluded from the analysis.

### Statistical analysis

Statistical analyses were performed using Python (Python Software Foundation), version 3.9, with the scipy, statsmodels, and pandas libraries. The normality of data distribution was assessed using the Shapiro-Wilk test and visual inspection of histograms.

Continuous demographic variables (age, total intracranial volume [TIV]) were compared among the three groups (HC, CD, ULD) using one-way analysis of variance (ANOVA), while categorical variables (sex) were analyzed using the Pearson Chi-squared test. Group differences in thalamic nuclei volumes were evaluated using analysis of covariance (ANCOVA). To account for potential confounding factors identified in the demographic analysis, age, sex, and TIV were included as covariates in the general linear model.

The main effect of “Group” was assessed for each thalamic nucleus. To control for Type I errors due to multiple comparisons across the analyzed subnuclei, the Benjamini-Hochberg False Discovery Rate (FDR) correction procedure was applied. Statistical significance was analysed by *p* -values adjusted for FDR (pFDR), which were reported alongside uncorrected values. Multiple comparison control (FDR) was applied simultaneously across all thalamic structures analysed (26 structures in total in both hemispheres).

Post-hoc pairwise comparisons (specifically HC vs. CD and HC vs. ULD) were conducted using independent sample t-tests for regions showing significant or trend-level main effects. To quantify the magnitude of volumetric differences independent of sample size, Cohen's d effect sizes were calculated. Effect sizes were interpreted as small (d = 0.2), medium (d = 0.5), and large (d ≥ 0.8).

Thalamic asymmetry was investigated by calculating an asymmetry index (AI) for each structure using the formula: *AI = (Right Volume- Left Volume)/(Right Volume + Left Volume)* . Group differences in AIs were analyzed using the same ANCOVA model described above.

Finally, to explore the relationship between thalamic structural integrity and clinical disease severity, Spearman's rank correlation coefficients (ρ, Rho) were calculated between BFMDRS scores and the combined bilateral volumes of the 13 thalamic subnuclei normalized by TIV. A two-tailed *p* -value < 0.05 was considered statistically significant for all analyses.

## RESULTS

### Demographic and clinical characteristics

The demographic and clinical characteristics of the study participants are summarized in Table 1 . The cohort consisted of 91 participants: 37 HC, 25 patients with CD, and 29 patients with ULD. A significant difference was observed in age among the groups (F = 12.89, *p* < 0.001), with the CD group being older than both the HC and ULD groups. Additionally, TIV differed significantly between groups ( *p* = 0.036). Therefore, all subsequent volumetric analyses were rigorously adjusted for age, sex, and TIV to control for these confounding factors. Sex distribution did not differ significantly between groups ( *X* ^2^ = 3.48, *p* = 0.176).

**Table 1:** Demographic and clinical characteristics of the study participants

Characteristics	HC (n = 37)	CD (n = 25)	ULD (n = 29)	*p* -value
Mean age (years)	40.83 ± 13.60	56.44 ± 11.51	44.13 ± 11.00	**< 0.001** a
Sex (female/male): n	23/14	22/3	24/5	0.176 b
Mean TIV (cm ^3^ )	1,450.2 ± 150.4	1,380.5 ± 120.1	1,410.8 ± 130.6	**0.036** a
Mean BFMDRS score	N/A	5.8 ± 3.1	4.9 ± 3.5	0.345 c

Abbreviations: BFMDRS, Burke-Fahn-Marsden dystonia rating scale; CD, cervical dystonia; HC, healthy controls; N/A, not applicable; TIV, total intracranial volume; ULD, Upper Limb Dystonia. Notes: ^a^ Calculated using One-way ANOVA. ^b^ Calculated using Pearson's Chi-squared test. ^c^ Calculated using independent samples t-test (CD vs. ULD). Bold values indicate statistical significance ( *p* < 0.05).

### Thalamic nuclei volumetry

**Figure 1:**
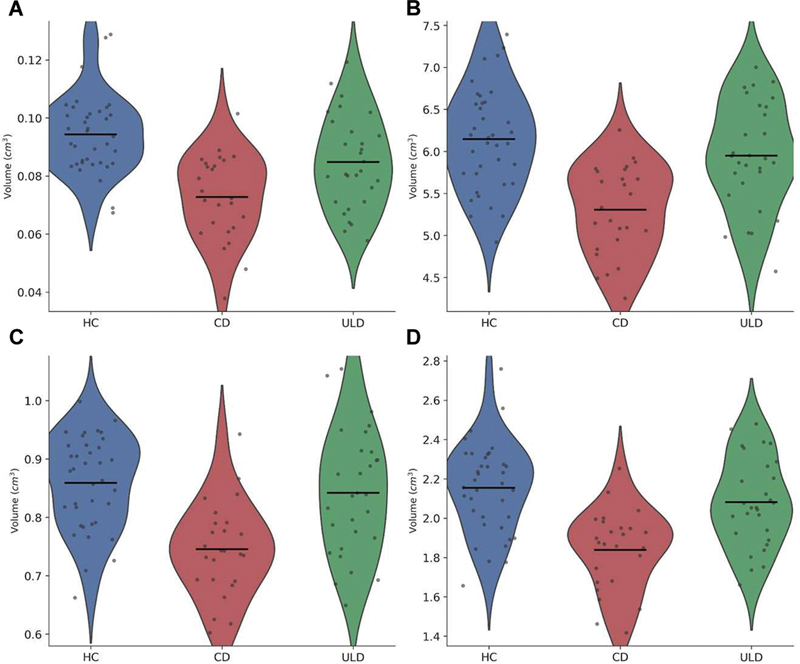
Abbreviations: CD, cervical dystonia; HC, healthy controls; ISN, intermediate space nuclei; LGN, lateral geniculate nuclei; ULD, upper limb dystonia; VLPN, ventral lateral posterior nuclei. Violin plots illustrating volumetric atrophy in specific thalamic nuclei across study groups. The violin shapes represent the probability density of the volume distribution, while the scattered black dots correspond to individual data points for each participant. The horizontal black bars indicate the group means. ( **A** ) Left LGN and ( **B** ) left whole thalamus show pronounced atrophy in the CD group (red) compared to HC (blue). ( **C** ) Right VLPN and ( **D** ) right ISN demonstrate significant volumetric reduction in CD patients, reflecting widespread involvement of motor and intralaminar thalamic circuits. Notably, the ULD group (green) shows relatively preserved volumes comparable to controls. Volumes are unadjusted raw values expressed in cm ^3^ .

The ANCOVA results revealed distinct patterns of thalamic atrophy, predominantly affecting the CD group ( Table 2 and Figure 1 ). These patients exhibited widespread volumetric reductions in comparison to HC. The most pronounced atrophy was observed in the left lateral geniculate nucleus (LGN; *p* = 0.003) and the left whole thalamus ( *p* = 0.006).

**Table 2:** Group comparisons of thalamic nuclei volumes adjusted for covariates

Structure (Nucleus)		Side	HC (Mean ± SD)	CD (Mean ± SD)	ULD (Mean ± SD)	ANCOVA *p*	FDR *p*	Cohen's d (HC-CD)
**Visual/Sensory nuclei**	LGN	L	0.094 ± 0.01	0.073 ± 0.02	0.085 ± 0.01	**0.003**	0.078	**1.55**
		R	0.096 ± 0.01	0.081 ± 0.02	0.091 ± 0.01	0.063	0.160	0.99
	MGN	L	0.081 ± 0.01	0.076 ± 0.01	0.083 ± 0.01	0.147	0.254	0.48
		R	0.079 ± 0.01	0.072 ± 0.01	0.077 ± 0.01	0.421	0.537	0.54
	PN	L	1.39 ± 0.16	1.19 ± 0.16	1.34 ± 0.14	**0.038**	0.133	**1.25**
		R	1.36 ± 0.14	1.21 ± 0.15	1.31 ± 0.14	0.085	0.187	1.05
**Motor nuclei**	VLPN	R	0.86 ± 0.09	0.75 ± 0.08	0.84 ± 0.09	**0.017**	0.078	**1.42**
		L	0.84 ± 0.09	0.74 ± 0.08	0.82 ± 0.09	**0.046**	0.149	**1.34**
	VAN	R	0.27 ± 0.04	0.23 ± 0.04	0.26 ± 0.04	**0.026**	0.099	0.87
		L	0.27 ± 0.04	0.26 ± 0.04	0.27 ± 0.04	0.407	0.534	0.36
	VLAN	R	0.10 ± 0.02	0.09 ± 0.02	0.10 ± 0.01	0.069	0.160	0.90
		L	0.10 ± 0.01	0.09 ± 0.01	0.10 ± 0.01	0.179	0.289	0.64
**Limbic/Intralaminar**	ISN	R	2.15 ± 0.24	1.84 ± 0.23	2.08 ± 0.20	**0.012**	0.078	**1.43**
		L	2.12 ± 0.25	1.81 ± 0.23	2.04 ± 0.22	**0.020**	0.086	**1.36**
	MTT	R	0.02 ± 0.01	0.02 ± 0.01	0.02 ± 0.01	0.244	0.354	0.48
		L	0.02 ± 0.01	0.02 ± 0.01	0.02 ± 0.01	0.056	0.154	0.82
	AVN	R	0.10 ± 0.02	0.09 ± 0.02	0.10 ± 0.02	0.261	0.364	0.54
		L	0.10 ± 0.02	0.09 ± 0.02	0.09 ± 0.01	0.490	0.593	0.53
	MN	R	0.63 ± 0.07	0.59 ± 0.07	0.62 ± 0.06	0.359	0.486	0.53
		L	0.63 ± 0.07	0.57 ± 0.08	0.62 ± 0.07	0.121	0.231	0.99
	Whole Thalamus	T	12.3 ± 1.25	10.6 ± 1.13	11.9 ± 1.21	**0.009**	0.078	**1.45**

Abbreviations: ANCOVA, analysis of covariance; AVN, anterior ventral nuclei; CD, cervical dystonia;CN, centromedian nuclei; Hb, habenular nuclei; HC, healthy controls; ISN, intermediate space nuclei; L, left; LGN, lateral geniculate nuclei; MGN, medial geniculate nuclei; MN, mediodorsal nuclei; MTT, mammillothalamic tract nuclei; PN, pulvinar nuclei; R, right; T, total; VAN, ventral anterior nuclei; VLAN, ventral lateral anterior nuclei; VLPN, ventral lateral posterior nuclei; VPLN, ventral posterior lateral nuclei. Notes: Volumes are expressed in cm ^3^ (mean ± standard deviation). Group differences were assessed using ANCOVA with age, sex, and TIV included as covariates. ANCOVA p: Uncorrected *p* -value for the main effect of group. FDR p: *p* -value corrected for multiple comparisons using the Benjamini-Hochberg False Discovery Rate method. Cohen's d: Effect size for the pairwise comparison between HC and CD patients. Bold values indicate *p* < 0.05 or large effect sizes (d ≥ 0.8). The table only includes the basic motor, sensory, and limbic nuclei.

Crucially, significant atrophy in the CD group extended to specific motor and intralaminar nuclei. The right ventral lateral posterior nucleus (VLPN), a key relay node in the cerebello-thalamo-cortical motor circuit, showed significant volume loss ( *p* = 0.017). Similarly, the right intermediate space (ISN; *p* = 0.012) and right ventral anterior nucleus (VAN; *p* = 0.026) were significantly smaller in CD patients compared to controls.

Although the *p* -values for these volumetric differences marginally exceeded the strict significance threshold after FDR correction ( *p _FDR_* = 0.078), the effect sizes for the CD group were remarkably large. Specifically, Cohen's d values exceeded 1.4 for the LGN, whole thalamus, VLPN, and ISN comparisons (HC vs. CD), suggesting a notable biological trend toward atrophy that warrants further investigation in larger cohorts, although these results should be interpreted with caution given the lack of strict statistical significance after FDR correction. In contrast, the ULD group showed relatively preserved thalamic volumes, with no significant volumetric differences in any thalamic nucleus found when compared to HC ( Table 2 ).

### Thalamic asymmetry

To determine whether the observed atrophy was lateralized, we analyzed the AIs of thalamic nuclei. As detailed in Table 3 , no statistically significant differences in asymmetry were found between groups for any of the analyzed structures ( *p* > 0.05). The lack of significant asymmetry in the CD group, despite the substantial volume loss, suggests that the neuroanatomical change process in CD affects both thalamic hemispheres symmetrically.

**Table 3:** Analysis of thalamic asymmetry indices

Structure	AI ANCOVA *p-* value	Interpretation
MGN	0.048	Trend-level difference
VAN	0.205	Symmetric
Whole thalamus	0.250	Symmetric
VLAN	0.290	Symmetric
MN	0.264	Symmetric
VLPN	0.714	Symmetric

Abbreviations: AI, asymmetry index; ANCOVA, analysis of covariance; MGN, medial geniculate nuclei; MN, mediodorsal nuclei; VAN, ventral anterior nuclei; VLAN, ventral lateral anterior nuclei; VLPN, ventral lateral posterior nuclei. Notes: AI was calculated as (Right - Left) / (Right + Left). Group differences in AI were analyzed using ANCOVA adjusted for age and sex. No significant differences survived multiple comparison correction, indicating bilateral involvement in the affected groups.

### Clinical correlations

**Figure 2:**
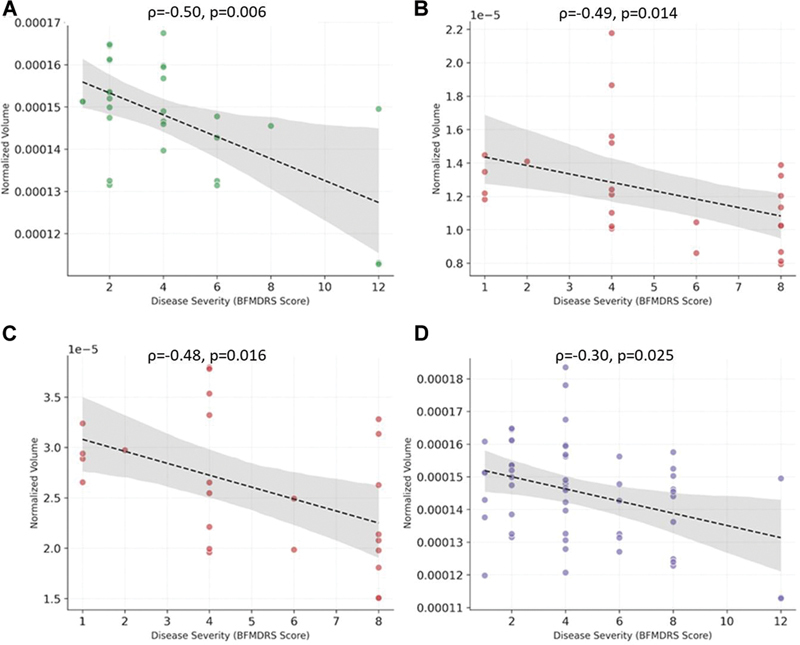
Abbreviations: AVN, anterior ventral nuclei; BFMDRS, Burke-Fahn-Marsden Dystonia Rating Scale; CD, cervical dystonia; CI, confidence interval; MTT, mammillothalamic tract nuclei; TIV, total intracranial volume. ULD, upper limb dystonia. Scatter plots showing significant correlations between clinical disease severity and normalized thalamic nuclei volumes. Relationships were assessed using Spearman's rank correlation analysis. ( **A** ) In patients with ULD, higher disease severity (BFMDRS score) is significantly associated with reduced volume of the AVN (ρ = −0.50, *p* = 0.006). ( **B** ) In patients with CD, higher disease severity correlates with atrophy of the right MTT (ρ = −0.49, *p* = 0.014). ( **C** ) This negative correlation in the CD group is also observed for the total MTT volume. ( **D** ) Across the entire patient cohort (CD + ULD), a negative association between disease severity and AVN volume is maintained. The solid lines represent the linear regression fit, and the shaded areas indicate the 95%CIs. Volumes are normalized by the TIV.

We investigated the relationship between disease severity, measured by the BFMDRS, and normalized thalamic volumes ( Table 4 and Figure 2 ). In the ULD group, a significant negative correlation was identified between disease severity and the volume of the anterior ventral nuclei (AVN; ρ = −0.50, *p* = 0.006), suggesting that greater atrophy in the AVN is associated with more severe clinical symptoms.

**Table 4:** Spearman correlation analysis between disease severity and normalized thalamic volumes

Groups	Thalamic nucleus	Spearman's Rho (ρ)	*p* -value
ULD	AVN	−0.50	**0.006**
CD	MTT	−0.49	**0.014**
All Patients (CD + ULD)	MTT	−0.32	**0.017**
All Patients (CD + ULD)	AVN	−0.30	**0.025**

Abbreviations: AVN, anterior ventral nuclei; MTT, mammillothalamic tract nuclei. Notes: Correlations were performed between the BFMDRS scores and thalamic volumes normalized by TIV. Significant negative correlations indicate that higher disease severity is associated with lower specific thalamic nuclei volumes. In these analyses, bilateral (right + left) total volume data were used for each nucleus.

In the CD group, disease severity was significantly negatively correlated with the volume of the MTT (ρ = −0.49, *p* = 0.013). These findings highlight distinct phenotype-specific structure-function relationships within the thalamus for different dystonia subtypes.

## DISCUSSION

This study investigated the volumetric characteristics of thalamic nuclei in patients with two different subtypes of idiopathic focal dystonia, CD and ULD, using the ultra-high-resolution MRI-based deep learning method (DeepThalamus). Our findings revealed a trend of volumetric reduction, particularly in specific thalamic subregions such as the left LGN, right VLPN, and right ISN in the CD group compared to healthy controls. Although the statistical significance remained at the borderline after multiple comparison corrections (p _*FDR*_ > 0.05), the substantial effect sizes in these regions (Cohen's d > 1.4) may suggest a potential role of thalamic microstructural changes in CD pathophysiology.

Furthermore, the negative correlations observed between disease severity and specific thalamic nucleus volumes (MTT in CD, AVN in ULD) support the hypothesis that thalamic degeneration could be associated with the clinical phenotype. However, given the limitation of the sample size, these findings should be interpreted as strong preliminary data for future studies rather than definitive pathological evidence.

The thalamus acts as a central “relay” station for transmitting motor signals from the basal ganglia and cerebellum to the cortex. Previous literature has reported thalamic volume loss and microstructural abnormalities in idiopathic dystonia. Xu et al. 9 demonstrated a decrease in bilateral thalamic gray matter volume in a large idiopathic dystonia cohort, correlating this atrophy with thalamocortical white matter degeneration. 9 The trend of thalamic volume reduction observed in the CD group in our study, although not statistically confirmed, is consistent with this literature. Particularly, the signal of volumetric reduction in the VLPN, a key node in motor control networks, may be associated with disruptions in the transmission of signals from the cerebellum to the motor cortex in the pathogenesis of dystonia, as emphasized by Lenz et al. 6

Another noteworthy finding of our study is the trend of decreased volume in the left LGN in the CD group. While the LGN is traditionally known as part of the visual pathways, recent studies emphasize the importance of sensory and visual integration deficits in dystonia. Xu et al. identified LGN atrophy in blepharospasm patients and linked it to visual sensory symptoms like photophobia commonly seen in these patients. 10 In CD patients, visual-spatial perception impairments and faulty modulation of neck movements based on visual references (including the sensory trick mechanism) are well-documented. 1 Therefore, LGN atrophy may represent a structural correlate of non-motor visual-sensory processing deficits in CD.

Our findings provide a critical extension to the results reported by Paulo et al., 8 who utilized the same dataset to highlight divergent network involvement in focal dystonia subtypes. While they analyzed the thalamus as a single entity and identified global thalamic atrophy specifically in CD—accompanied by widespread white matter changes—our subnuclear analysis offers a more granular perspective. 8 By employing the DeepThalamus pipeline, the present study demonstrates for the first time that this observed atrophy in CD is driven by volumetric reduction trends in specific motor (VLPN, VAN) and sensory/limbic (LGN, ISN) subregions. Conversely, the relative preservation of thalamic subnuclei in our ULD group complements Paulo et al.'s finding of a striatal compartment (matrix/striosome) imbalance in the same patients. This suggests that ULD pathophysiology may be more localized to striatal circuit reorganization, whereas CD involves a more extensive network degeneration that includes critical thalamic relay nodes. 8 Thus, our study adds significant novelty by pinpointing the exact thalamic substructures involved, reinforcing the idea of distinct neuroanatomical profiles across different dystonia phenotypes.

The concept that distinct focal dystonia phenotypes are associated with divergent neural network involvement is robustly supported by existing morphometric and functional literature. For example, Ramdhani et al. 14 conducted a comparative analysis revealing that task-specific forms, such as hand dystonia, primarily affect sensorimotor control regions including the primary somatosensory cortex, striatum, and cerebellar lobules VI to VIIa. In contrast, phenotypes like CD exhibit more localized gray matter alterations in distinct cerebellar areas, such as the left lobule VIIa.

Similarly, Berman et al. 15 identified that CD is characterized by microstructural impairments in the right cerebellum and left caudate, presenting a pattern that is functionally distinct and non-overlapping with the involvement of the globus pallidus and red nucleus observed in other focal forms like blepharospasm. This phenotype-specific segregation is further corroborated by recent investigations into deep brain stimulation networks.

Butenko et al. 16 demonstrated that clinical efficacy in treating cervical symptoms is mediated through the cingulo-opercular network—specifically linked to cerebellothalamic tracts and thalamic motor nuclei—whereas limb dystonia correlates with the basal ganglia circuits and somatotopic regions of the primary motor cortex. These insights are highly congruent with our observation of a thalamic atrophy trend specifically in the CD group.

Such findings reinforce the hypothesis that CD pathophysiology involves a more extensive and potentially degenerative network disruption, encompassing the thalamus and cerebellothalamocortical pathways, distinct from the more localized circuit disturbances seen in ULD.

When examining the relationship between BFMDRS score and thalamic volumes, a negative correlation was found between the score and MTT volume in the CD group. A significant component of the limbic system (Papez circuit), MTT plays a role in emotional/memory processes. It is well known that CD patients have a high prevalence of non-motor symptoms such as anxiety, depression, and pain, and their contribution to the disease burden is well recognized. 17 This finding suggests that the severity of motor symptoms in CD may parallel structural alterations in limbic circuits. However, it is unclear whether this correlation represents a causal relationship or a secondary effect related to the chronic course of the disease.

The main limitation of our study is the small sample size. As Kilic-Berkmen et al. have pointed out, focal dystonias are a heterogeneous group, requiring large cohorts for clear subtype distinctions. 18 Despite observing high effect sizes in our study, the lack of statistical significance (Type II error risk) necessitates a cautious interpretation of the findings. Therefore, rather than claiming thalamic atrophy as a definitive biomarker for CD, positioning this as a hypothesis-generating study for larger-scale, multicenter, longitudinal research would be more appropriate. Additionally, although the DeepThalamus method used for thalamic nucleus segmentation is highly sensitive, the resolution limits of 3T MRI images may lead to partial volume effects, especially in volumetric measurements of small nuclei (e.g., intralaminar nuclei).

Finally, correlation analyses examining the structure function relationships between thalamic subnucleus volumes and clinical disease severity were conducted using an exploratory approach. To avoid sacrificing findings of potential clinical importance, a strict FDR correction was not applied in these analyses. Therefore, the correlation results should be interpreted cautiously, as hypothesis-generating data for future studies rather than definitive evidence.

In conclusion, this study provides preliminary evidence that thalamic nuclei, particularly the VLPN and sensory/limbic (LGN, MTT) subregions, may be volumetrically affected in idiopathic CD. The absence of a similar atrophy pattern in the ULD group supports the idea that different neuroanatomical bases may underlie focal dystonia phenotypes. Future research should elucidate the functional implications of these structural changes and the role in disease progression using larger samples and multimodal imaging techniques (combined analyses with fMRI, DTI).
